# Altered immunoreactivity of ErbB4, a causative gene product for ALS19, in the spinal cord of patients with sporadic ALS

**DOI:** 10.1111/neup.12558

**Published:** 2019-05-24

**Authors:** Yuji Takahashi, Akiko Uchino, Ayako Shioya, Terunori Sano, Chihiro Matsumoto, Yurika Numata‐Uematsu, Seiichi Nagano, Toshiyuki Araki, Shigeo Murayama, Yuko Saito

**Affiliations:** ^1^ Department of Neurology, National Center Hospital National Center of Neurology and Psychiatry Kodaira Japan; ^2^ Department of Neurology and Neuropathology and Brain Bank for Aging Research Tokyo Metropolitan Geriatric Hospital and Institute of Gerontology Tokyo Japan; ^3^ Department of Laboratory Medicine, National Center Hospital National Center of Neurology and Psychiatry Kodaira Japan; ^4^ Tokyo Medical and Dental University Graduate School of Medical and Dental Sciences Tokyo Japan; ^5^ Department of Peripheral Nervous System Research, National Institute of Neuroscience National Center of Neurology and Psychiatry Kodaira Japan; ^6^ Department of Pediatrics Tohoku University School of Medicine Sendai Japan; ^7^ Department of Neurology Osaka University Graduate School of Medicine Osaka Japan

**Keywords:** ErbB4, immunohistochemistry, *SOD1*, sporadic amyotrophic lateral sclerosis, TDP‐43

## Abstract

ErbB4 is the protein implicated in familial amyotrophic lateral sclerosis (ALS), designated as ALS19. ErbB4 is a receptor tyrosine kinase activated by its ligands, neuregulins (NRG), and plays an essential role in the function and viability of motor neurons. Mutations in the ALS19 gene lead to the reduced autophosphorylation capacity of the ErbB4 protein upon stimulation with NRG‐1, suggesting that the disruption of the NRG–ErbB4 pathway causes motor neuron degeneration. We used immunohistochemistry to study ErbB4 in the spinal cord of patients with sporadic ALS (SALS) to test the hypothesis that ErbB4 may be involved in the pathogenesis of SALS. ErbB4 was specifically immunoreactive in the cytoplasm of motor neurons in the anterior horns of the spinal cord. In patients with SALS, some of the motor neurons lost immunoreactivity with ErbB4, with the proportion of motor neurons with a loss of immunoreactivity correlated with the severity of motor neuron loss. The subcellular localization was altered, demonstrating nucleolar or nuclear localization, threads/dots and spheroids. The ectopic glial immunoreactivity was observed, mainly in the oligodendrocytes of the lateral columns and anterior horns. The reduction in the ErbB4 immunoreactivity was significantly correlated with the cytoplasmic mislocalization of transactivation response DNA‐binding protein 43 kDa (TDP‐43) in the motor neurons. No alteration in immunoreactivity was observed in the motor neurons of mice carrying atransgene for mutant form of the superoxide dismutase 1 gene (*SOD1*). This study provided compelling evidence that ErbB4 is also involved in the pathophysiology of SALS, and that the disruption of the NRG–ErbB4 pathway may underlie the TDP‐43‐dependent motor neuron degeneration in ALS.

## INTRODUCTION

Amyotrophic lateralsclerosis (ALS) is a devastating neurological disorder characterized by progressive motor neuron degeneration resulting in generalized muscle weakness and atrophy with 2–4 years of the average life expectancy from the onset. Familial ALS (FALS) comprises 5–10% of ALS cases, while sporadic ALS (SALS) comprises the remaining 90–95%.^1^ To date, more than 20 genes have been shown to be implicated in FALS.^2^ The identification of FALS genes has been shown to contribute greatly to the understanding of the pathogenesis of not only FALS but also SALS.

*ERBB4* has been identified as a novel causative gene for autosomal dominant, late‐onset FALS, designated as ALS19.^3^ Patients with pathogenic mutations showed typical clinical features of ALS, clinically undistinguishable from those observed in patients with SALS. Functional analysis of mutations at the ALS19 locus revealed reduced autophosphorylation of the ErbB4 proteins upon stimulation by one of its ligands, neuregulin (NRG)‐1, suggesting that the disruption of the NRG–ErbB4 pathway leads to motor neuron degeneration. ErbB4 is a member of the epidermal growth factor (EGF) receptor subfamily of transmembrane receptor tyrosine kinases.^4^ Upon binding with NRG, it forms a homodimer or heterodimer with other subfamily members such as ErbB2 or ErbB3, thereby activating the autophosphorylation of its tyrosine kinase and C‐terminal domains to mediate a wide variety of downstream signaling cascades such as the phosphatidyinositide 3‐kinase‐Akt and Ras–mitogen‐activated protein kinase pathways.^5^, ^6^ The ErbB4 has splicing isoforms at the exons encoding the transmembrane domain, designated as JM‐a and JM‐b, and those encoding the C‐terminal domain, designated as CYT‐1 and CYT‐2,^7^, ^8^ which regulate the cellular survival, proliferation, differentiation or death, in a cell‐ and isoform‐specific manner.^9^, ^10^ The JM‐a isoform is cleaved by membrane‐bound proteases, including tumor growth factor‐α‐converting enzyme and γ‐secretase,^11^ followed by the translocation of its intracellular domain to the nucleus, thus directly acting as a transcriptional coregulator.

Previous studies have suggested that ErbB4 is one of the essential molecules in the function and viability of motor neurons: ErbB4 is abundantly expressed in rat spinal motor neurons^12^ and also localized at neuromuscular junctions,^13^ where the NRG–ErbB4 signaling pathway plays an important role in the development and modulation of the neuromuscular junction.^14^, ^15^ ErbB4 is one of the important signaling proteins in C‐bouton synapses on somatic motor neurons.^16^ Aberrant NRG–ErbB signaling has been implicated in the spinal motor neurons of FALS, SALS and mouse models with *SOD1* mutations.^17^, ^18^ These findings raise the possibility that ErbB4 is involved in the pathogenesis not only of ALS19, but also of ALS in general. Herein, we performed immunohistochemical (IHC) studies on ErbB4 in the spinal cord of patients with SALS to investigate the relevance of ErbB4 in the pathogenesis of SALS.

## MATERIALS AND METHODS

### Patients

The National Center of Neurology and Psychiatry (NCNP) Brain Bank and the Brain Bank for Aging Research (BBAR) in the Tokyo Metropolitan Geriatric Hospital and Institute of Gerontology (TMGHIG) have been established to promote pathological studies of neurological disorders. Among the autopsy specimens registered in these brain banks, we included 18 patients with a confirmed clinical diagnosis of ALS based on the El Escorial and Airlie House revised criteria (11 males and seven females). The average age of onset, age at autopsy and the disease duration were 67.4 ± 6.3 years (range, 55–80), 70.9 ± 5.8 years (range, 56–83) and 29.8 ± 15.8 months (range, 5–85), respectively. A patient with a 5‐month disease duration died due to respiratory insufficiency complicated by pulmonary asbestosis, presenting with mild right‐predominant weakness in the upper extremities and no obvious weakness in the lower extremities. We also included 14 control subjects without neurological diseases with an average age at autopsy of 72.8 ± 4 years (range, 63–82), and four disease controls consisting of two patients with spinobulbar muscular atrophy (SBMA), one with myasthenia gravis (MG) complicated with gastric cancer, and one with multiple system atrophy (MSA). Written informed consent was obtained from all patients or their next to kin. This study was performed in keeping with the code of ethics of the World Medical Association (Declaration of Helsinki) as approved by the institutional review boards of NCNP and TMGHIG.

### Autopsy tissue preparation, staining and IHC

The autopsy specimens were processed according to the BBAR Brain Bank protocol as previously described.^19^ Briefly, at the time of autopsy, half of the brain was preserved at −80°C, and the other half was fixed in 20% neutral‐buffered formalin (Wako, Osaka, Japan) for 7–13 days and then sectioned. Representative areas of the brain and spinal cord were embedded in paraffin and cut serially into 6‐μm‐thick sections. For the autopsies conducted prior to April 2011, spinal cords were fixed with 20% formalin, while spinal cords sampled from April 2011 were fixed in 4% paraformaldehyde in 0.1 M phosphate buffer (pH 7.4) for 48 h. IHC analysis for ErbB4 was performed using the Ventana Discovery XT automated IHC/ISH research slide staining system (Roche, Basel, Switzerland) according to the manufacturer's instructions. Briefly, the specimens were deparaffinized, pretreated with citrate buffer (pH 6.0), and autoclaved for 20 min at 121°C. The commercially available rabbit polyclonal antibody against ErbB4 (sc‐283 (C‐18); Santa Cruz Biochemistry, Dallas, TX, USA), in which antibody specificity has been established through reabsorption analysis,^12^ was applied as a 1:400 dilution for the primary antibody reaction at 37°C for 32 min, followed by a biotin‐conjugated antirabbit secondary antibody reaction at 37°C for 16 min. The sections were developed with the streptavidin‐biotin complex method using the Ventana I‐VIEWDAB Universal Kit (Roche). Immunostaining using antibodies for TDP‐43 or phosphorylated TDP‐43 was performed as previously described.^20^ Likewise, double immunostaining was performed with sc‐283 at a dilution of 1:500, rabbit polyclonal antibody against TDP‐43 (10782‐2‐AP; Proteintech, Rosemont, IL, USA) at dilution of 1:5000, rabbit polyclonal antibody against ionized Ca^2+^‐binding adaptor molecule 1 (Iba1) (019‐19741; Wako Chemicals, Osaka, Japan) at a dilution of 1:2000, or rabbit polyclonal antibody against glial fibrillary acidic protein (GFAP) (N1506; Dako, Glostrup, Denmark) using the Ventana Discovery/XT automated IHC/ISH research slide staining system and the DISCOVERY RedMap Kit (Roche). Histological sections were observed under a research microscope (Eclipse 90i; Nikon, Tokyo, Japan). Neuropathological findings were assessed according to a semiquantitative rating scale based on the following points: motor neuron loss, TDP‐43 pathology (dystrophic neurites, glial cytoplasmic inclusions, neuronal cytoplasmic inclusions) as previously described,^20^ number of motor neurons with loss of ErbB4 immunoreactivity, nuclear immunoreactivity, threads/dots, spheroids/globoids, gliosis and glial immunoreactivity. These findings were graded as severe (3), moderate (2), mild (1) or negative (0).

### Animals

We used transgenic mice harboring the high‐copy G93A‐mutated form of the human superoxide dismutase 1 gene (*SOD1*) (C57Bl/6J‐TgN[SOD1‐G93A]1Gur) originally purchased from the Jackson Laboratory (Bar Harbor, ME, USA) and backcrossed with C57Bl/6J mice. The mice were anesthetized with diethyl ether at day 113 or 140, and perfusion fixation was performed with 4% paraformaldehyde using a peristaltic pump with a flush line inserted in the right atrium. C57Bl/6J mice of the same age were also used for control experiments. All the animal experiments were approved by the Animal Care Committee of NCNP.

### Statistical analysis

The correlation between ErbB4 immunoreactivity and TDP‐43 pathology in motor neurons was statistically analyzed using the χ^2^‐test. Results with *P*‐values less than 0.05 were considered statistically significant.

## RESULTS

### ErbB4 immunoreactivity in normal subjects and disease controls

**Figure 1:**
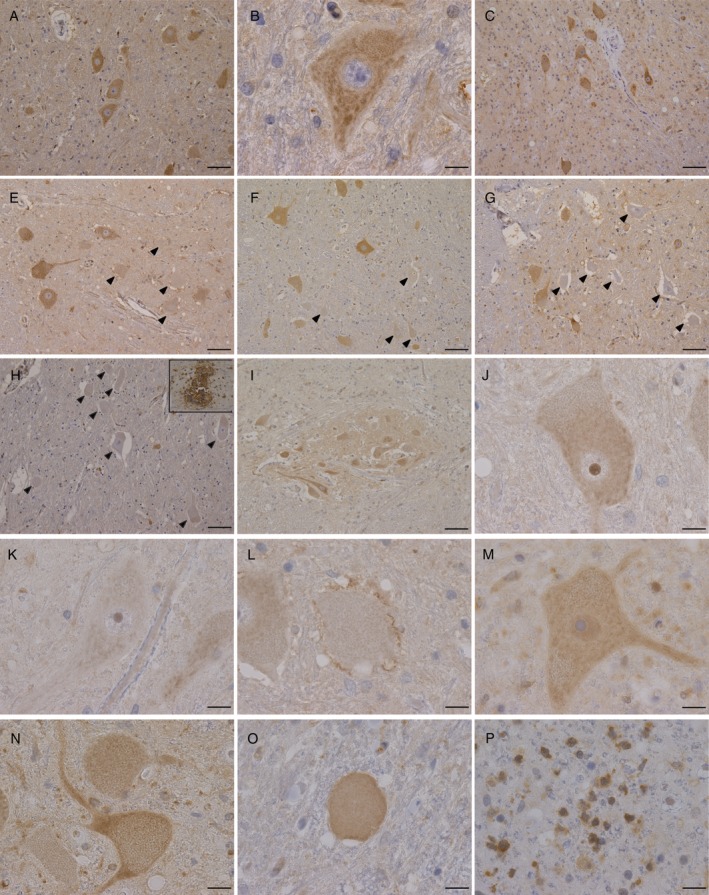
Microphotographs of GrbB4‐immunostained spinal cord sections from a normal control (A, B), an SBMA patient (C), an MSA patient (D), and SALS patients (E‐P). Immunoreactivity is observed in the cytoplasm of motor neurons in normal and disease controls (A‐D), but undetectable in motor neurons (arrowheads) in SALS patients with different disease durations (E: 5 months; F: 12 months; G: 24 months; H: 36 months). Ependymal cells show retained immunoreactivity (H, inset). Motor neurons in Onuf's nucleus show retained immunoreactivity (I). Immunoreactivity is prominent in the nucleoli (J, K) and tiny, thread‐ or granule‐like in glialcells surrounding a motor neuron, which lacks immunoreactivity in a patientwith a 5‐month disease duration (L). Note that the immunoreactivity is reduced in the cytoplasm (K). Immunoreactivity is dense in the nucleus but not in thenucleolus in patients described above (M). Dense immunoreactivity appearingthread‐ and dot‐like structures is observed in the axon hillock and the proximaldendrites (N). Immunoreactivity is detectable in a spheroid (O) and glial cellsin the lateral column (P). Scale bars: 100 μm (A, C‐I), 20 μm (B, J‐P).

In the spinal cords derived from normal subjects, ErbB4 immunoreactivity was observed in the motor neurons of the anterior horns (Fig. 1A). In addition, the substantia gelatinosa and ependymal cells surrounding the central canal were also immunostained: the latter were used as internal controls. No immunoreactivity was observed in the white matter of the spinal cord. The immunoreactivity was diffusely located in the cytoplasm of the motor neurons, with dense granular immunoreactivity scattered predominantly in the vicinity of the cellular membrane (Fig. 1B). No immunoreactivity was observed in the nucleus, while its adjacent cytoplasmic area showed relatively reduced immunoreactivity.

The immunostaining pattern was similar in the disease controls. Particularly, the immunoreactivity was retained in the cytoplasm of the residual motor neurons in patients with SBMA, as observed in normal controls (Fig. 1B). No glial staining was recognized. The immunoreactivity patterns were normal in MSA (Fig. 1C) and in MG.

### Dysregulation of ErbB4 immunoreactivity in the motor neurons of patients with SALS

In patients with SALS, some of the residual motor neurons exhibited a loss of immunoreactivity. These neurons were intermingled with neurons showing normal or slightly enhanced immunoreactivity in the same section (Fig. 1E–H). There was a tendency that the proportion of motor neurons with a loss of immunoreactivity was corelated with the severity of motor neuron loss, although some of the sections exhibited profound loss of immunoreactivity even when the motor neuron loss was mild. It was also noted that the shrinkage of cell bodies was more obvious in motor neurons with a loss of immunoreactivity. In the advanced stages, a majority of the residual motor neurons lost its ErbB4 immunoreactivity, whereas the immunoreactivity of the ependymal cells was retained (Fig. 1H). In contrast, motor neurons in the Onuf's nuclei invariably retained immunoreactivity in the cytoplasm (Fig. 1I).

The subcellular localization was altered in some of the residual motor neurons. In a patient with a 5‐month disease duration, prominent nucleolar immunoreactivity was observed in some of the motor neurons (Fig. 1J). These neurons showed decreased immunoreactivity in the cytoplasm (Fig. 1K). Tiny, thread‐ or granule‐like immunoreactive patches were frequently observed in the areas surrounding the motor neurons with a loss of immunoreactivity. These were considered to be synaptic terminals projecting to the neurons (Fig. 1L). In addition, some sections derived from patients with SALS showed diffuse, dense immunoreactivity in the nuclei as well as in the cytoplasm of motor neurons. These neurons tended to show enhanced immunoreactivity in the cytoplasm (Fig. 1M). In contrast to that observed in a patient with a very early age of onset, nucleolar immunostaining was not remarkable in these neurons. The immunoreactivity in axons, axon hillocks or proximal dendrites, presenting as threads or dots was also observed (Fig. 1N). Motor neurons with dense nuclear immunoreactivity tended to coexist with threads or dots (Table 1). Spheroids or globoids were also intensely immunostained (Fig. 1O). Glial immunoreactivity was observed in the anterior horns, anterior columns or lateral columns in SALS (Fig. 1P). Glial immunoreactivity was observed in most of the patients, although several specimens did not show obvious glial immunoreactivity, despite remarkable gliosis (Table 1). Glial immunoreactivity in the posterior columns was inconspicuous compared with that in other regions. No evidence of colocalization of ErbB4 immunoreactivity with skein‐like inclusions, Lewy body‐like inclusions or Bunina bodies was obtained in this study.

**Table 1:** Summary of pathological findings in the spinal cords from SALS patients

①	②	③	④	⑤	⑥	⑦	⑧	⑨	⑩			⑪	⑫	⑬	⑭	⑮
									DN	GCI	NCI					
1	M	80	80	5	U	L	0	1	1	1	1	1	1	2	2	1
						C	1	2	1	1	3	2	0	2	1	2
						C	2	3	1	1	3	3	0	1	0	3
2	M	65	66	12	L	L	1	2	3	1	2	1	0	2	2	1
						C	2	2	N.A.	N.A.	N.A.	2	0	1	1	1
3	F	70	71	12	B	L	1	2	2	1	3	2	0	1	2	0
						C	2	2	3	2	3	2	0	2	2	1
4	M	62	64	16	U	L	3	2	N.A.	N.A.	N.A.	3	0	0	0	2
						C	3	1	0	2	2	3	0	0	0	1
5	F	75	77	21	B	L	3	2	N.A.	N.A.	N.A.	3	0	1	1	2
6	M	70	72	24	D	L	1	1	1	1	2	2	2	2	2	1
						C	1	2	N.A.	N.A.	N.A.	2	0	2	1	1
7	M	72	74	24	U	L	3	1	1	2	2	3	0	0	1	1
						C	3	1	N.A.	N.A.	N.A.	3	0	1	1	1
8	F	55	56	24	L	L	2	3	3	3	2	2	0	0	1	3
						C	3	3	2	3	2	3	0	0	1	3
9	F	73	75	24	U	L	1	1	2	2	2	1	1	2	1	1
10	M	75	77	26	U	C	3	2	1	3	2	3	0	0	0	1
11	M	64	67	28	N.D.	L	2	3	0	3	3	3	1	3	0	3
						C	3	3	0	3	2	3	1	3	0	3
12	F	80	83	30	U	L	1	2	1	2	3	1	3	2	1	2
13	M	58	61	31	U	L	2	1	0	2	2	2	0	0	0	0
						C	1	1	1	2	1	1	1	0	1	0
14	F	67	70	36	U	L	2	2	1	1	1	2	1	1	1	1
						C	3	2	N.A.	N.A.	N.A.	3	1	1	1	1
15	F	76	79	36	D	L	1	2	1	1	3	3	0	1	2	2
						C	1	2	1	1	3	3	0	1	3	2
16	M	71	74	39	L	L	3	2	0	3	1	3	0	0	0	2
						C	3	1	0	3	1	3	0	0	0	2
17	M	60	65	59	U	C	1	1	1	1	2	1	0	0	0	0
18	M	58	66	85	L	Th	3	2	0	2	0	3	0	1	2	2
						C	3	2	1	3	0	3	0	0	0	2

Neuropathological findings were assessed based on a semiquantitative rating scale graded as severe (3), moderate (2), mild (1) and negative (0).

Subheadings: ① patient, ② sex ③ age at onset, ④ age at autopsy, ⑤ duration (months) of disease, ⑥ onset site (U: upper extremity, L: lower extremity, B: bulbar, D: dementia), ⑦ regions examined (C: cervical. Th: thoracic, L: lumbar), ⑧ motor neuron loss, ⑨ gliosis, ⑩ TDP‐43 pathology, ⑪–⑮ immunoreactivity of ErbB4, ⑪ proportion of motor neurons with loss of immunoreactivity of ErbB4, ⑫ nuclear immunoreactivity, ⑬ threads/dots, ⑭ spheroids/globoids, ⑮ glial immunoreactivity.

DN, dystrophic neurites; GCI, glial cytoplasmic inclusions; N.A., not analyzed; N.D., not described; NCI, neuronal cytoplasmic inclusions.

### Correlation between ErbB4 immunoreactivity and subcellular localization of TDP‐43 in spinal motor neurons

**Figure 2:**
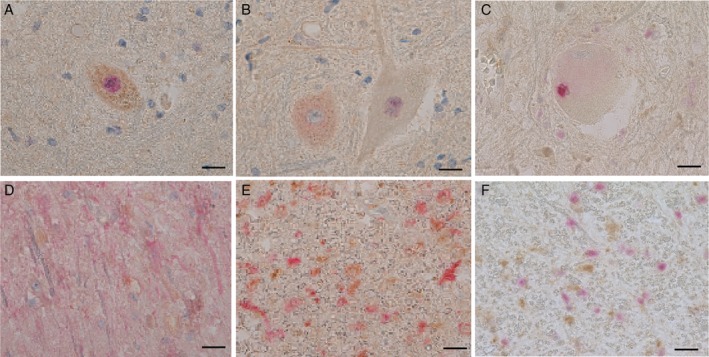
Microphotographs of double‐immunostained spinal cord sections from SALS patients (A‐C, F: ErbB4 (brown) and TDP‐43 (red); ErbB4 (brown) and GFAP (red); E: ErbB4 (brown) and Iba1 (red)). ErbB4 immunoreactivity in motor neurons is localized at TDP‐43‐positive nucleus (A), TDP‐43‐positive cytoplasm (B), TDP‐43‐negative nucleus (B), or TDP‐43‐positive cytoplasmic inclusions (C). ErbB4‐positive glial cellsare negative for GFAP (D), Iba1 (E), or TDP‐43 (F). Scale bars: 20 μm (A‐F).

Comparison of the number of motor neurons showing loss of ErbB4 immunoreactivity with the grading of TDP‐43 pathology raised the possibility that these findings were correlated with each other (Table 1). To address this possibility, double immunostaining of ErbB4 and TDP‐43 was conducted in the spinal cords derived from three patients with SALS; we observed that motor neurons with retained or lost immunostaining coexisted (Fig. 2A–E). There were five types of motor neurons classified according to the immunoreactivity of ErbB4 and the subcellular localization of TDP‐43: positive ErbB4 immunoreactivity with nuclear localization of TDP‐43 (Fig. 2A) or with diffuse cytoplasmic localization of TDP‐43 (Fig. 2B), and negative ErbB4 immunoreactivity with nuclear localization of TDP‐43 (Fig. 2B), with diffuse cytoplasmic localization of TDP‐43 or with cytoplasmic inclusions of TDP‐43 (Fig. 2C). In total, 91 motor neurons were manually counted (Table 2). The loss of ErbB4 immunoreactivity was significantly correlated with the subcellular localization of TDP‐43 (χ^2^ = 13.42, *P* = 0.0012). Of note, all of the motor neurons with cytoplasmic aggregations of TDP‐43 showed loss of ErbB4 immunoreactivity. Twenty motor neurons with nuclear localization of TDP‐43 also showed loss of ErbB4 immunoreactivity, implying that the dysregulation of ErbB4 expression could occur prior to the cytoplasmic mislocalization of TDP‐43. In contrast, all of the neurons with enhanced immunoreactivity of ErbB4 showed nuclear localization of TDP‐43.

**Table 2:** The number of motor neurons classified based on the immunoreactivity of ErbB4 and the subcellular localization of TDP‐43

		Immunoreactivity of ErbB4		
		Positive	Negative	Total
Subcellular localization of TDP‐43	Nuclear	18	20	38
	Cytoplasmic (diffuse)	8	31	39
	Cytoplasmic (inclusions)	0	14	14
	Total	26	65	91

The motor neurons were counted manually and classified according to the immunoreactivity of ErbB4 (positive or negative) and the subcellular localization of TDP‐43 (nuclear localization, cytoplasmic localization with diffuse immunostaining or cytoplasmic localization with inclusions).

### Characterization of ErbB4‐positive glial cells

To characterize the ErbB4‐positive glial cells, double immunostaining of ErbB4 and GFAP (Fig. 2D) or Iba1 (Fig. 2E) was performed. The ErbB4‐positive glial cells did not show positive GFAP or Iba1 immunoreactivity, raising the possibility that the ErbB4 immunoreactivity was localized in the oligodendrocytes. Double immunostaining of ErbB4 and TDP‐43 in the lateral columns tended to show reciprocal patterns between ErbB4‐positive and TDP‐43‐positive glial cells: those with ErbB4 immunoreactivity did not show TDP‐43 immunoreactivity and vice versa (Fig. 2F).

### ErbB4 immunoreactivity in mutant *SOD1* transgenic mice

**Figure 3:**
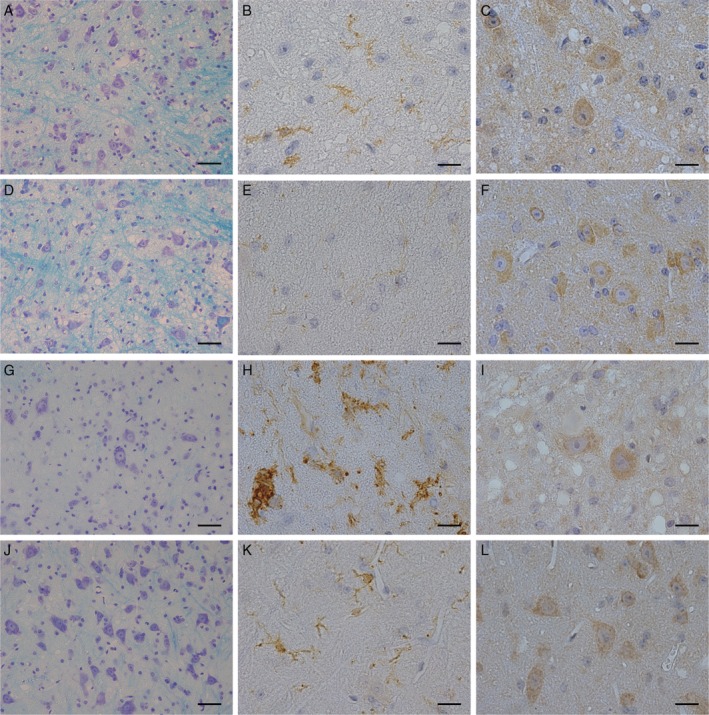
Microphotographs of spinal cord sections from mutant *SOD1* transgenic mice (A‐C, G‐I) and nontransgenic littermates (D‐F, J‐L) at early symptomatic stage (A‐F: day 113) and end stage (G‐L: day 140), stained with KB (A, D, G, J) as well as immunostained for Iba1 (B, E, H, K) and ErbB4 (C, F, I, L). Scale bars: 20 μm (A‐L).

Immunohistochemistry was performed in mutant *SOD1* transgenic (Tg) mice (Fig. 3A–C, G–I) and their wild‐type (WT) littermates (Fig. 3D–F, J–L) at day 113, the early symptomatic stage (Fig. 3A–F), or at day 140, the end stage (Fig. 3G–L).^21^ At day 113, moderate loss of motor neurons (Fig. 3A) and gliosis with mild microglial activation were observed in the Tg mice (Fig. 3B) compared with that in WT mice (Fig. 3D,E). The ErbB4 immunoreactivity was retained in the cytoplasm of motor neurons in Tg (Fig. 3C) to the similar level as WT (Fig. 3F) mice. At day 140, 50% of the motor neurons were lost (Fig. 3G), while prominent proliferation of the activated microglia was observed in the spinal cords of the Tg mice (Fig. 3H), which was not observed in the WT (Fig. 3J,K). At this stage, ErbB4 immunoreactivity was also retained in the cytoplasm of residual motor neurons of the Tg mice (Fig. 3I) to the similar level as WT (Fig. 3L) mice. Neither nucleolar nor diffuse nuclear immunoreactivity was observed in the motor neurons of Tg mice. No glial immunoreactivity corresponding to microglial activation was detected in the Tg mice.

## DISCUSSION

This study demonstrated that extensive dysregulation of ErbB4 immunoreactivity was observed in the spinal cords of patients with SALS. Particularly, the loss of immunoreactivity in the motor neurons was prominent in some of the residual neurons, the proportion of which increased with an increase in the disease duration. In contrast, residual motor neurons in SBMA or mutant *SOD1* mice at the advanced disease stages showed retained immunoreactivity in the cytoplasm of the motor neurons, indicating that the dysregulation was not merely a secondary phenomenon following motor neuron dysfunction. The immunohistochemistry results in mutant *SOD1* transgenic mice were different from those observed in a previous study showing reduced immunoreactivity in the residual motor neurons, which may be due to differences in the epitopes of the antibodies: an antibody raised against the C‐terminal domain of ErbB4 was used in this study, in contrast to the antibody against the N‐terminal extracellular domain that was used in the previous study.^22^

It is reasonable to consider the hypothesis that the reduced expression of ErbB4 should deteriorate the function and accelerate the degeneration of motor neurons in patients with SALS, considering that the disruption of the NRG–ErbB4 pathway is assumed to be an essential mechanism in ALS19. This hypothesis is further supported by a number of animal model studies demonstrating that aberrant NRG–ErbB4 signaling causes motor neuron dysfunction and degeneration.^23^, ^24^, ^25^, ^26^, ^27^ Interestingly, the motor neurons showing a loss of immunoreactivity were intermingled with motor neurons with retained or enhanced immunoreactivity. We speculate that the enhanced ErbB4 immunoreactivity observed in some motor neurons may compensate for the degenerating motor neurons.

The correlation between the loss of ErbB4 immunoreactivity and the cytoplasmic localization of TDP‐43 indicates that ErbB4 is involved in the TDP‐43‐dependent pathophysiology of ALS. Some of the motor neurons with nuclear localization of TDP‐43 exhibited loss of ErbB4 immunoreactivity, suggesting that the nuclear functions of TDP‐43 in terms of ErbB4 expression may already be perturbed prior to the initiation of cytoplasmic mislocalization. The retained immunoreactivity in the motor neurons of mutant *SOD1* mice is consistent with this notion as the mutant *SOD1*‐dependent pathophysiology has been presumed to be distinct from the TDP‐43‐dependent pathophysiology.^28^ TDP‐43 is a RNA‐binding protein and its target RNA molecules have been extensively investigated.^29^, ^30^ *ERBB4* mRNA has been identified as one of the binding targets for TDP‐43, raising the possibility that TDP‐43 mislocalization may affect the RNA metabolism of *ERBB4*. Recently, TDP‐43 has been shown to control endosomal trafficking of ErbB4 in neurons.^31^ The study demonstrates that TDP‐43 knockdown decreased the surface expression of ErbB4, resulting in reduced dendrite complexity in neurons. The reduction in dendrite complexity was completely rescued with ErbB4 expression, suggesting that ErbB4 is one of the key effector molecules in TDP‐43‐dependent motor neuron dysfunction.^31^

Dynamic alteration of localization of ErbB4 immunoreactivity was observed in the motor neurons of patients with SALS; the following are of particular interest: (i) localization in the nucleolus; (ii) immunoreactivity in spheroids; and (iii) ectopic immunoreactivity in oligodendrocytes. First, the nucleolar localization of ErbB4 has been widely investigated in cancer cells, in which the C‐terminal region of ErbB4 binds to nucleolin, a principal component of the nucleolus that regulates differentiation and proliferation.^32^, ^33^ Nucleolar stress is an emerging hypothesis to explain motor neuron degeneration caused by the hexanucleotide repeat expansion of *C9ORF72*, which has been shown to bind to nucleolin in the nucleolus and causes nucleolar stress.^34^ Poly Gly‐Arg/Pro‐Arg dipeptide repeats produced by repeat‐associated non‐ATG (RAN) translation of C9ORF72 localized in the nucleolus caused nucleolar stress resulting in aberrant RNA metabolism^35^ or impaired stress granule formation,^36^ both of which have been implicated in ALS pathogenesis. Based on these findings, it seems plausible that ErbB4 could also be involved in nucleolar stress by binding to nucleolin in the initial phases during the pathophysiology of SALS. Second, axonal spheroids containing abnormally accumulated neurofilaments is a major cytopathological hallmark of ALS pathology in humans and mouse models, and is thought to contribute to the selective vulnerability of long, large‐caliber motor axons.^37^, ^38^, ^39^ Causative gene products for ALS such as *SOD1* 40 or peripherin^38^ have been identified as components of spheroids in addition to neurofilaments. Identification of ErbB4 as a novel component of spheroid indicates the molecular convergence of causative genes for ALS, corroborating the vital role of impaired axonal transport and the relevance of ErbB4 in the pathophysiology of ALS. Third, ectopic immunoreactivity of ErbB4 in oligodendrocytes was another aspect of dysregulation unraveled in this study. The ErbB4 immunostaining pattern observed was in sharp contrast to that of ErbB2, its family member protein and potential counterpart to form heterodimers; ErbB2 immunoreactivity was noted in activated microglia.^18^ ErbB4 is an important regulator of oligodendrocyte development and maturation,^10^, ^41^, ^42^, ^43^ and controls the myelin formation by oligodendrocytes in the CNS.^44^ An important role of oligodendrocytes is one of the emerging concepts in the pathogenesis of ALS, as evidenced by IHC studies demonstrating oligodendrocyte degeneration and TDP‐43 pathology in the spinal cords derived from patients with SALS.^45^, ^46^, ^47^ The reciprocal immunoreactivity patterns of ErbB4 and TDP‐43 observed in this study raises the possibility that the expression of ErbB4 and TDP‐43 may be mutually exclusive in oligodendrocytes. Dysregulation of ErbB4 may provide a clue to investigate the role of oligodendrocytes in the pathophysiology of ALS.

In conclusion, this study provided compelling evidence that ErbB4 is also involved in the pathophysiology of SALS. Disentangling the complex aspects of the dysregulated ErbB4 expression by clarifying the underlining mechanisms would further deepen our understanding of the pathophysiology of ALS. Elucidating versatile roles of ErbB4 in ALS pathophysiology may provide us with ideas for the development of innovative disease‐modifying therapies for ALS such as modulation of ErbB4 functions by NRG or their analogs.
